# Supplementary material for: How to Engage Health Care Workers in the Evaluation of Hospitals: Development and Validation of BSC-HCW1—A Cross-Sectional Study
Source: Int J Environ Res Public Health. 2022 Jul 26;19(15):9096. doi: 10.3390/ijerph19159096 (PMC9367997; doi:10.3390/ijerph19159096)
Supplement: Supplementary file 1 [file ijerph-19-09096-s001.zip › ijerph-1778724-supplementary.pdf]

Supplementary Materials:

Table S1. The items that did not load in EFA.

Code	Question
ESMO3	I receive recognition for the work that is well done from the superiors
EST2	This hospital provides me with the needed updates on how to use the technology/information system
ESE1	My performance is assessed periodically
ESR3	I belief that this hospital has better reputation than other hospitals in Palestinian
ESD2	The hospital provides me education on infection control and safety measures

Table S2. The items that were suggested to be deleted based on Cattell's scree plot analysis in EFA.

Code	Question
ESR2	I belief that patients respect health care workers at this hospital and trust them
ESR4	I am proud to work with this hospital
ESA2	It is easy to have access to the hospital when a case is urgent
ESC4	Communication with management is frequent and they keep me updated with sufficient information to do my job
ESMO1	I believe that the strategic goals of the hospital including mission, vision, aims, objectives in relationship with action plans are clear to me
ESC5	I trust what my direct manager tells me or promises me with
ESEM2	New Employees receive initial orientation period at the beginning of their work
ESEM1	New Employees are well introduced about job description and specification are clear in the job contract
ESF4	My direct superiors explain and discuss the strengths and weaknesses in my assessment with me
ESS1	When errors are reported a blame free policy is taken by managers
ESS2	Safety standards are implemented and assured (masks, gloves, sanitizers, etc.)
